# Circular RNA CDR1as/ciRS-7– a novel biomarker in solid tumors

**DOI:** 10.3389/fonc.2024.1468363

**Published:** 2024-11-29

**Authors:** Yun Zhang, Chanyu Xiong, Zhilin Jiang, Xiao Wang, Juanjuan Ji, Yan Pan, Tianshu Yu, Zihao Wang, Lin Zhu, Yumei Yue, Qiong Li, Haizhen Wang, Shikai Zhu, Yu Zhou

**Affiliations:** ^1^ Sichuan Provincial Key Laboratory for Human Disease Gene Study, Genome Sequencing Center, Department of Laboratory Medicine, Sichuan Provincial People’s Hospital, School of Medicine, University of Electronic Science and Technology of China, Chengdu, China; ^2^ Organ Transplant Center, Sichuan Provincial Key Laboratory for Clinical Immunology Translational Medicine, School of Medicine, Sichuan Provincial People’s Hospital, University of Electronic Science and Technology of China, Chengdu, China; ^3^ Department of Cell and Molecular Pharmacology & Experimental Therapeutics, Hollings Cancer Center, Medical University of South Carolina, Charleston, SC, United States

**Keywords:** circular RNA, CDR1as/ciRS-7, cancer, prognosis, meta-analysis, solid tumors

## Abstract

**Introduction:**

Circular RNA CDR1as/ciRS-7 has been reported to function as an oncogenic regulator in various cancers. However, the prognostic value of CDR1as/ciRS-7 expression in solid tumors remains unclear. Herein, we conducted an updated meta-analysis to investigate the association between CDR1as/ciRS-7 expression and clinical outcomes in solid tumors.

**Methods:**

A systematic search was performed through the PubMed, EMBASE, Web of Science, and Ovid databases for eligible studies on clinical values of CDR1as/ciRS-7 in solid tumors. The pooled hazard ratios (HRs) or odd ratios (ORs) with 95% confidence intervals (CIs) were used to evaluate the correlation between CDR1as/ciRS-7 and clinical outcomes.

**Results:**

A total of 2424 patients from 17 studies between 2017 and 2023 were included. The results suggested that elevated CDR1as/ciRS-7 expression predicted a poor overall survival (OS) for 12 types of solid tumors (HR=1.93, 95% CI: 1.43-2.60, P<0.001) with no heterogeneity (I2 = 80.2%, P<0.001). Stratified analysis indicated that there was a negative relationship between CDR1as/ciRS-7 expression and OS in digestive system cancers (HR=2.30, 95% CI: 1.84-2.88, P<0.001), and respiratory cancers (HR=2.40, 95% CI: 1.75-3.30, P<0.001). Furthermore, we also revealed that CDR1as/ciRS-7 was positively related to tumor size (OR=2.11, 95%CI: 1.64-2.71, P<0.001), TNM stage (OR=2.05, 95%CI: 1.65-2.54, P<0.001), lymph node metastasis (LNM) (OR=1.74, 95%CI: 1.38-2.21, P<0.001), and distant metastasis (OR=2.79, 95%CI: 1.71-4.55, P<0.001). Although the probable evidence of publication bias was found in the studies with OS, tumor size, TNM stage, and LNM, the trim and fill analysis confirmed the reliability of these results was not affected.

**Conclusion:**

Elevated CDR1as/ciRS-7 expression was associated with larger tumor size, advanced TNM stage, worse LNM, distant metastasis, and shorter OS, suggesting that CDR1as/ciRS-7 may act as an independent prognostic biomarker in solid tumors.

## 1 Introduction

Cancer is a human complex genetic disease, and it is one of the crucial public health problems worldwide (1). With the raising of cancer incidence and mortality, cancer has become the leading cause of death since 2010 in China (2). Despite great advances in the diagnosis and treatment of cancers, the clinical prognosis of cancer patients is still poor. Therefore, the development of early detection and novel therapeutic methods based on the elucidation of the molecular pathogenesis of human cancers are urgently needed.

Circular RNAs (circRNAs) are one new kind of lncRNAs, which have no 5’ or 3’ ends but are covalently linked to form a closed circular structure (3). Increasing evidence suggest-ed that circRNAs could regulate gene expression at the transcriptional or posttranscriptional level through binding to miRNAs or other molecules. And circRNAs play crucial roles in multiple human diseases, such as diabetes, arteriosclerosis, cardiac hypertrophy, and cancer (4–10). Numerous circRNAs have been identified as regulators of cancer development and even treatment (11).

Cerebellar degeneration-related protein 1 antisense RNA (CDR1as) is a highly conserved circRNA. It contains more than 70 repetitive miR-7 binding sites that function as miR-7 sponges**;** hence, it is also called ciRS-7 (12). The biological function of miR-7 is abolished by the overexpression of CDR1as/ciRS-7, which results in decreased miR-7 activity and elevated expression of miR-7 targeting genes (13, 14). By sponging miR-7, CDR1as/ciRS-7 disrupts its regulatory functions, which can lead to the dysregulation of various target genes involved in cell proliferation, apoptosis, and differentiation (15, 16). To be specific, studies demonstrate that CDR1as/ciRS-7 is abnormally expressed in many different types of solid tumors, including cholangiocarcinoma (CCA) (17), colorectal cancer (CRC) (18, 19), non-small cell lung cancer (NSCLC) (20–22), larynx-geal squamous cell carcinoma (LSCC) (23), esophageal squamous cell carcinoma (ESCC) (24, 25), breast cancer(BrC) (26), gastric cancer (GC) (27, 28), melanoma (29), nasopharyngeal carcinoma (NPC) (30), ovarian cancer (OC) (31), clear cell renal cell carcinoma (ccRCC) (32), and cervical cancer (CC) (33).

As a cancer-related circRNA, increasing evidence supports that CDR1as/ciRS-7 may serve as a negative prognostic biomarker in various cancers, and high CDR1as/ciRS-7 ex-pression correlated with larger tumor size, advanced TNM stage, worse lymph node metastasis and distant metastasis (34–37). However, to the best of our knowledge, most studies reported on the prognostic role of CDR1as/ciRS-7 expression are limited by sample size and discrete clinical outcomes. And circRNAs such as CDR1as/ciRS-7 have not been put into practice as biomarkers in clinical decision-making, and proper validation studies involving prospectively collected samples and clinical trials are lacking. Thus, in this study, we conducted a systematic review and quantitative meta-analysis to investigate the clinicopathological and prognostic value of CDR1as/ciRS-7 as a potential biomarker in human solid tumors.

## 2 Methods

### 2.1 Search strategy and study selection

We searched through the PubMed, EMBASE, Web of Science, and Ovid databases for potentially eligible studies on clinical values of CDR1as/ciRS-7 expression in human solid tumors from inception up to June 2024. The search terms were included: “circular RNA”, “CDR1as” or “ciRS-7”, “cancer” or “tumor” or “carcinoma” or “neoplasm”. The reference lists of the retrieved studies were searched manually, and the literature search was per-formed by two independent researchers (Yun Zhang and Shikai Zhu).

The studies were considered eligible if they met the following criteria: any type of human cancer was studied; the studies investigated the prognostic value of CDR1as/ciRS-7 expression in cancers; the levels of CDR1as/ciRS-7 expression in cancerous tissues were detected; patients were grouped according to the levels of CDR1as/ciRS-7 expression; the studies included an association between CDR1as/ciRS-7 and clinicopathologic parameters; the studies provided sufficient data to estimate the HRs with corresponding 95% CI for OS; and the studies were published in English. The exclusion criteria were as follows: letters, editorials, expert opinions, case reports and reviews; the studies only investigated the molecular structure and functions of CDR1as/ciRS-7; the studies did not include the usable data for further analysis; and duplicate publications.

### 2.2 Data extraction and quality assessment

Two researchers (Yun Zhang and Chanyu Xiong) independently evaluated and extracted the eligible research data from each study, and the third researcher (Shikai Zhu) achieved a consensus for disagreements. The following elements were extracted from the Included studies: first author, publication date, country, tumor type, TNM stage, sample size, cut-off value, follow-up period, detection method, adjuvant therapy before the surgery, survival analysis methodology, HRs with corresponding 95% CIs for OS, disease-free survival (DFS) and progression−free survival (PFS), and other clinicopathologic parameters including age, gender, tumor size, tumor differentiation, TNM stage, lymph node metastasis and distant metastasis. HRs with corresponding 95% CIs were preferentially extracted from univariate or multivariate analyzes. If the data was not available, we calculated the HRs from Kaplan-Meier survival curves using Engauge Digitizer V4.1 software.

We assessed the quality of included studies based on the Newcastle-Ottawa scale (NOS) criteria. The NOS criteria use a “star” rating system ranging from 0 to 9 stars for the judgment of methodological quality, which was based on selection (0-4 stars), comparability (0-2 stars), and outcome (0-3 stars). Studies with more than 5 points were considered to be high quality. The quality of each study was independently evaluated by two afore-mentioned researchers. Inconsistent evaluations or data in the included studies were re-solved by discussion with the third investigator (Shikai Zhu).

### 2.3 Statistical analyses

STATA 14.0 statistical software (Stata Corporation, College Station, TX, USA) was used to analyze all the data. The pooled HRs with 95% CIs were used to estimate the prognostic value of CDR1as/ciRS-7 on OS, DFS and PFS in patients with solid tumors. Pooled ORs with 95% CIs were used to evaluate the relationship between CDR1as/ciRS-7 and clinicopathological characteristics of patients such as age, gender, tumor size, tumor differentiation, TNM stage, lymph node metastasis and distant metastasis. X2-based Cochran Q test and Higgins I2 statistic were utilized to analyze the heterogeneity among studies. If P-value <0.05 in combination with I2-value >50%, it was able to be considered significant heterogeneity. Random-effects models were used in cases which with significant heterogeneity. Subgroup analysis and sensitivity analysis were applied to dissect the heterogeneity. In addition, Begg’s funnel plot and Egger’s linear regression test were used to determine publication bias. Trim and fill analysis were performed if there was the possible evidence of publication bias. P-value <0.05 was considered statistically significant.

## 3 Results

### 3.1 Study selection and characteristics

**Figure 1:**
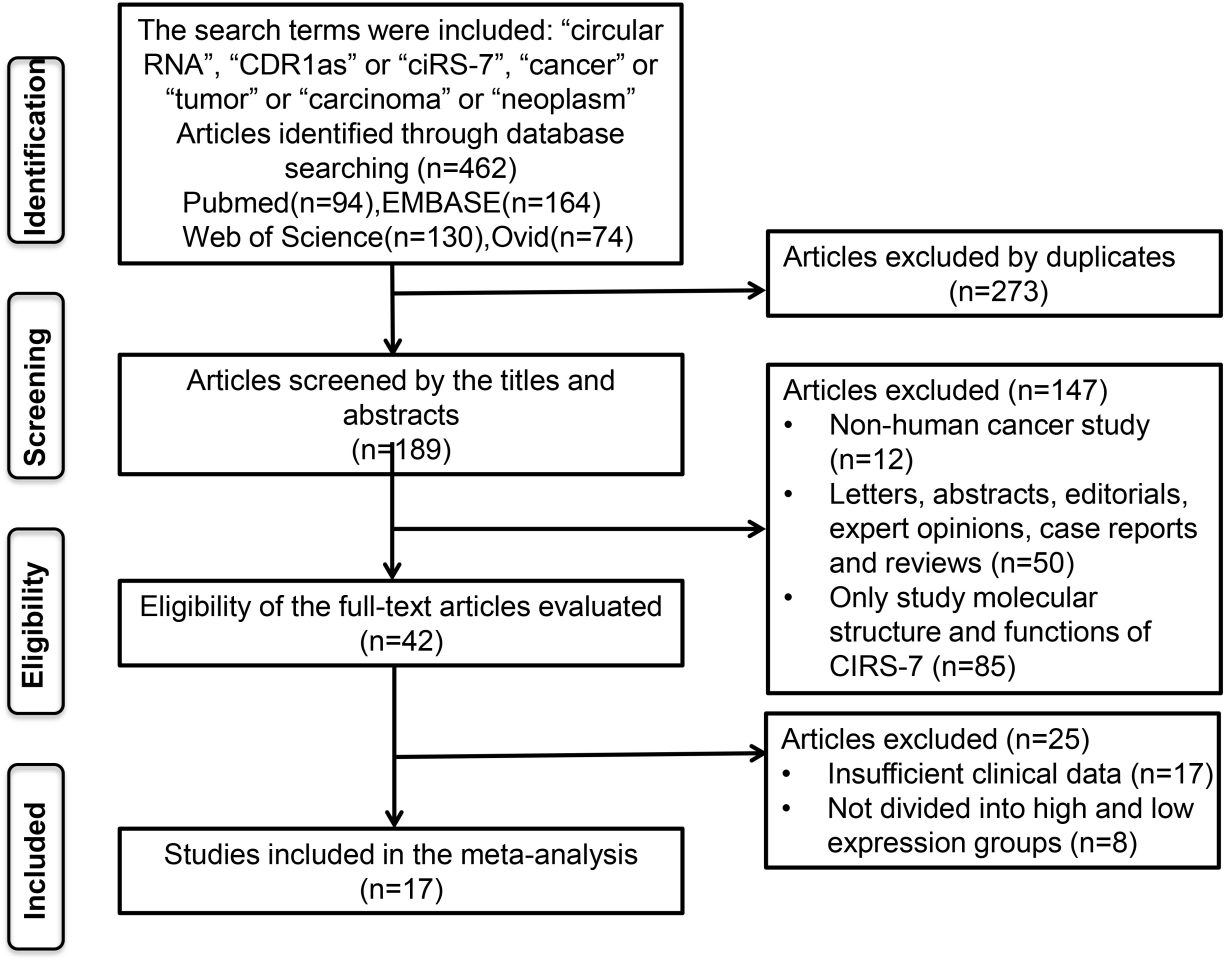
The flow diagram of this meta-analysis.

A total of 462 potentially relevant studies were identified in this meta-analysis. To achieve relevant studies, we evaluated the titles, abstracts, and author information of all collected articles, and 273 duplicate studies were excluded. After screening the titles and abstract carefully, 147 irrelevant studies, such as letters, editorials, expert opinions, case reports, reviews, and other types of uninvolved publications for the analysis and full-text review, were excluded. Through evaluating the eligibility of full-text articles, 25 articles without sufficient data or without dividing into high and low-expression groups were excluded. Finally, 17 eligible studies were included in this meta-analysis (**Figure 1**).

A total of 2424 patients from 17 studies (two of the studies each contributed two separate datasets) from 2017 to 2023 were included (**Table 1**) (17–33). Those studies were derived from China (n=14), Japan (n=1), Netherlands (n=1), and Spain (n=1). Among those studies, the sample size ranged from 30 to 352 patients, and more than 100 patients were enrolled in 9 studies. Twelve types of solid tumors, including CRC (n=2), CCA (n=1), GC (n=2), NSCLC (n=3), LSCC (n=1), ESCC (n=2), BrC (n=1), Melanoma (n=1), NPC (n=1), OC (n=1), ccRCC(n=1) and CC (n=1), were analyzed. The levels of CDR1as/ciRS-7 expression were measured by quantitative real-time polymerase chain reaction qRT-PCR (n=17) in all the studies. None of the patients received adjuvant therapy before the surgery in 17 studies. Multivariate analysis was included in 7 studies. Clinical outcomes were analyzed, including 16 studies for OS, 1 for DFS, and 1 for PFS. HRs with the corresponding 95% CIs for OS were extracted from the original data in 9 studies, and calculated from Kaplan-Meier Curves in other 8 studies (**Table 1**). Clinicopathologic parameters were also analyzed in 15 studies including age, gender, tumor size, tumor differentiation, TNM stages, LNM and distant metastasis (**Table 2**). Additionally, the studies with more than 6 according to the NOS score criteria were included to make sure the quality of the analysis (**Table 1**).

**Table 1:** The main characteristics of the included studies in the meta-analysis.

First author	Year	Region	Tumor Type	TNM Stage	Sample Size	Cut-off Value	Follow-up (months)	Detection Method	Adjuvant therapy	Survival Analysis	HR statistic	Outcome Measure	NOS
Jiang XM	2017	China	CCA	I-IV	54	Median	50 (total)	qRT-PCR	None	U/M	reported	OS	9
Weng WH	2017	China	CRC	II-IV	153	Median	44.4 (median)	qRT-PCR	None	U/M	reported	OS	6
	2017	Japan	CRC	II-IV	165	Median	61.2 (median)	qRT-PCR	None	U/M	reported	OS	9
Su CY	2017	China	NSCLC	I-IV	128	Mean	60 (total)	qRT-PCR	None	U/M	reported	OS	9
Tang WT	2017	China	CRC	I-IV	182	Median	60 (total)	qRT-PCR	None	U	reported	OS	9
Pan HY	2018	China	GC	II-IV	102	Median	60 (total)	qRT-PCR	None	U	calculated	OS	6
	2018	China	GC	II-IV	154	Median	60 (total)	qRT-PCR	None	U	calculated	OS	6
Zhang JZ	2018	China	LSCC	NA	30	NA	60 (total)	qRT-PCR	NA	U	calculated	OS	6
Li RC	2018	China	ESCC	I-III	123	Median	100 (total)	qRT-PCR	None	U	calculated	OS/DFS	7
Uhr K	2018	Netherlands	BrC	NA	345	Median	91 (median)	qRT-PCR	None	U	reported	OS	9
Yan B	2018	China	NSCLC	NA	132	Median	46 (median)	qRT-PCR	None	U/M	reported	OS	9
Zhang XF	2018	China	NSCLC	I-IV	60	Median	100 (total)	qRT-PCR	None	U/M	reported	OS	9
Sang MX	2018	China	ESCC	NA	86	Median	NA	qRT-PCR	None	NA	NA	NA	6
Zhong Q	2019	China	NPC	I-IV	44	Mean	100 (total)	qRT-PCR	NA	U	calculated	OS	8
Hanniford D	2020	Spain	Melanoma	NA	105	Quarter	175 (total)	qRT-PCR	NA	U	calculated	OS/MFS	6
Zhang FH	2020	China	OC	I-IV	40	NA	80 (total)	qRT-PCR	NA	U	calculated	OS	6
Zhao YH	2020	China	ccRCC	I-IV	87	Median	100 (total)	qRT-PCR	NA	U	calculated	PFS	9
Zhou Y	2020	China	CC	I-II	352	Median	60 (total)	qRT-PCR	None	U/M	calculated	OS	9
Li R	2023	China	GC	I-IV	82	NA	35.0 (median)	qRT-PCR	None	U/M	reported	OS	6

CCA, cholangiocellular carcinoma; CRC, colorectal cancer; NSCLC, non-small cell lung cancer; GC, gastric carcinoma; LSCC, lung squamous cell cancer; ESCC, esophageal squamous cell carcinoma; BrC, breast cancer; NPC, Nasopharyngeal carcinoma; OC, Ovarian cancer; ccRCC, clear cell renal cell carcinoma; CC, cervical cancer; U, univariate analysis; M, multivariate analysis; OS, overall survival; DFS, disease free survival; MFS, Metastasis-Free Survival.

**Table 2:** Correlation between CDR1as/ciRS-7 expression and clinicopathological characteristics of cancer.

Clinical parameters	No. of studies	No. of patients	OR (95% CI)	*P*-value	Heterogeneity	
					*I^2^*	*P*-value
Age (elder *vs.* younger)	12	1516	1.00(0.81-1.23)	0.990	0.0	0.78
Gender (male *vs.* female)	11	1156	0.83(0.65-1.06)	0.135	3.0	0.41
Tumor size (larger *vs.* smaller)	10	1372	2.11(1.64-2.71)	<0.001	0.0	0.54
TNM stage (III+IV *vs.* I+II)	12	1518	2.05(1.65-2.54)	<0.001	21.4	0.23
Lymph node metastasis (present *vs.* absent)	9	1205	1.75(1.38-2.21)	<0.001	62.4	0.01
Distant metastasis (present *vs.* absent)	7	780	2.79(1.71-4.55)	<0.001	0.0	0.44
Tumor differentiation (poor *vs.* well)	10	1380	1.99(1.49-2.68)	<0.001	51.1	0.03

### 3.2 Prognostic value of CDR1as/ciRS-7 expression in solid tumor

**Figure 2:**
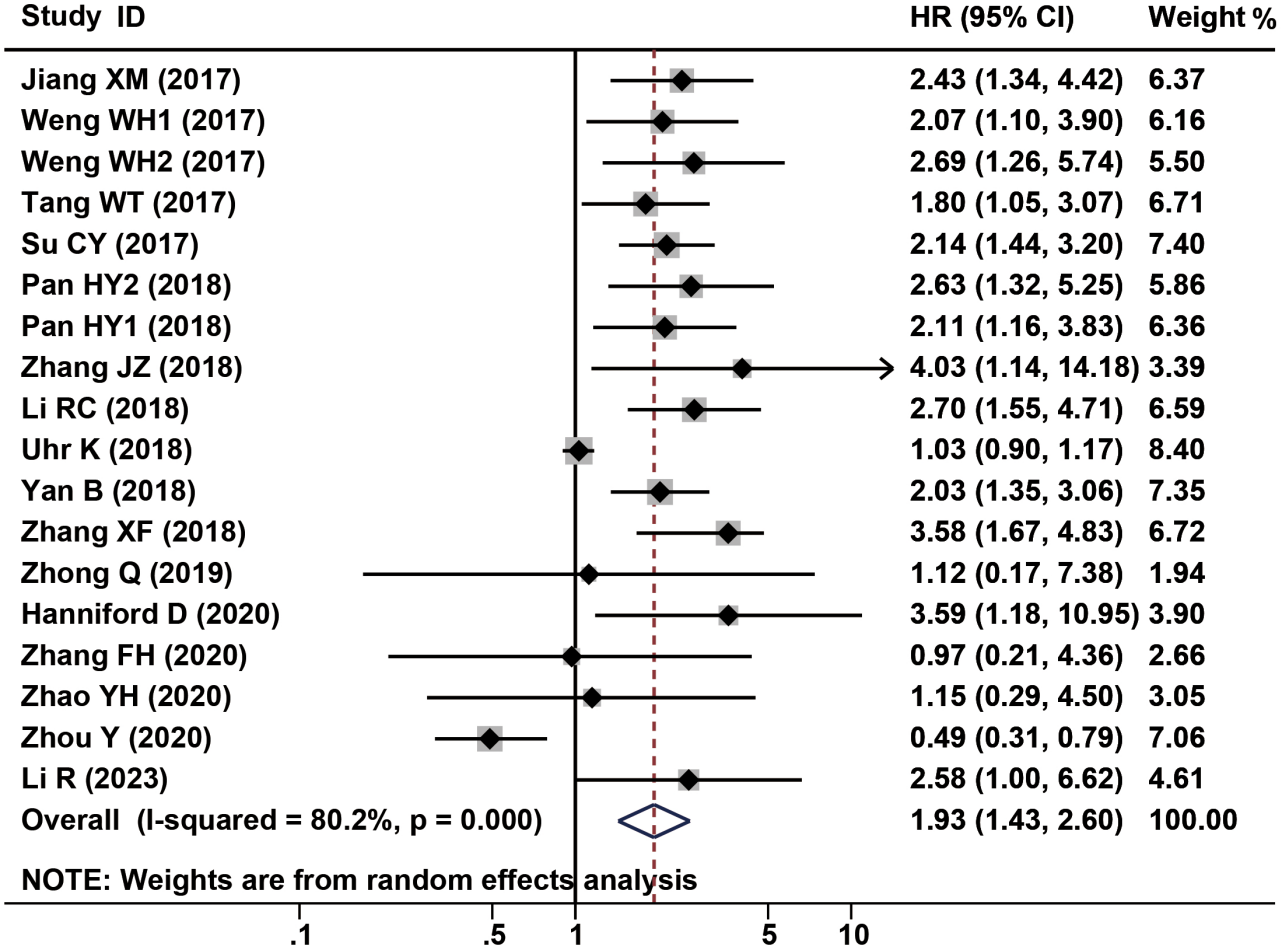
Forest plots of the HRs for the association between CDR1as/ciRS-7 expression and OS.

**Figure 3:**
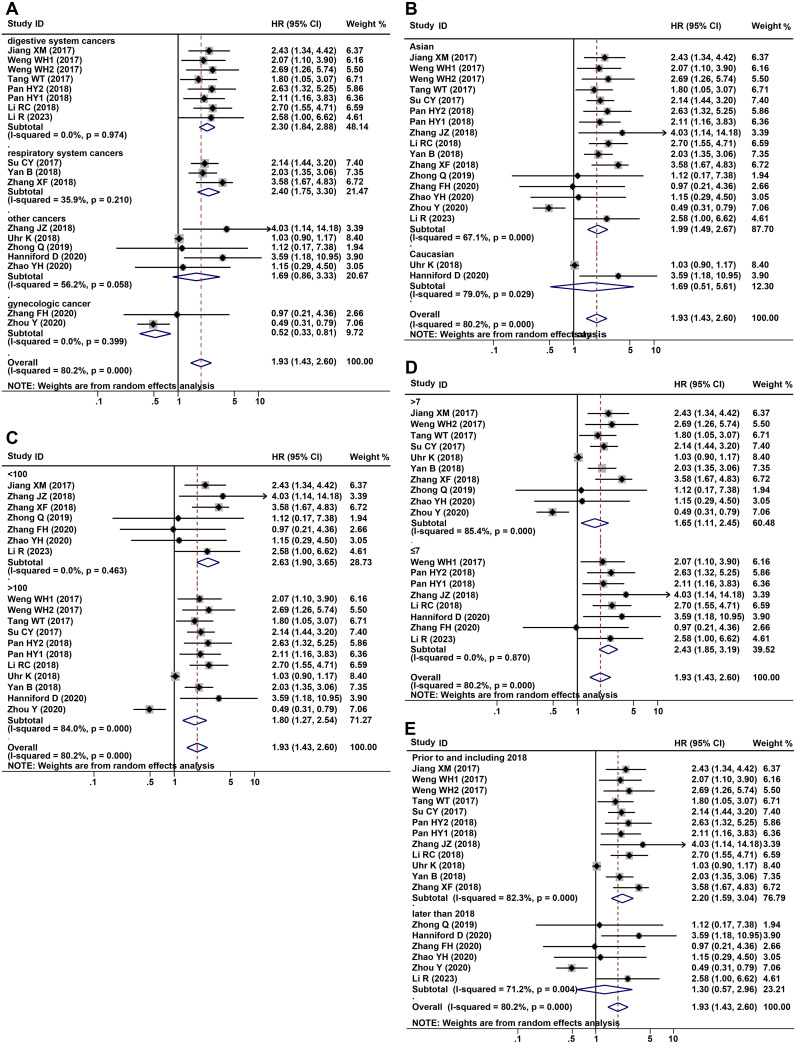
Forest plots of subgroup analysis for the HRs of OS by **(A)** cancer type, **(B)** detection method, sample size **(C)**, NOS **(D)** and year of public **(E)**.

2338 patients from a total of 16 studies were applied to assess the prognostic of CDR1as/ciRS-7 on OS in human solid tumors. The results suggested that elevated CDR1as/ciRS-7 expression predicted a poor OS for 12 types of solid tumors (HR=1.93, 95% CI: 1.43-2.60, P<0.001) with significant heterogeneity (I^2^ = 80.2%, P<0.001) (**Figure 2**). Furthermore, subgroup analysis was also conducted to investigate the association between HRs and cancer type/ethnicity/sample size/NOS. Stratified analysis indicated that there was a negative relationship between CDR1as/ciRS-7 and OS in the studies with digestive system cancers (HR=2.30, 95% CI: 1.84-2.88, P<0.001), and respiratory cancers (HR=2.40, 95% CI: 1.75-3.30, P<0.001). However, the higher expression of CDR1as/ciRS-7 predicted better OS in gynecological system cancers (HR=0.52, 95% CI: 0.33-0.81, P=0.004) (**Figure 3A**). And we found that upregulation of CDR1as/ciRS-7 expression significantly correlated with short OS in patients from Asian (HR=1.99, 95% CI: 1.49-2.67, P<0.001), while this correlation does not exist in Caucasian patients (HR=1.69, 95% CI: 0.51-5.61, P=0.389) (**Figure 3B**). Higher CDR1as/ciRS-7 expression predicted shorter OS in the studies with sample size >100 (HR=1.80, 95% CI: 1.27-2.54, P=0.001) as well as those with sample size <100 (HR=2.63, 95% CI: 1.90-3.65, P<0.001) (**Figure 3C**). In addition, the effect of CDR1as/ciRS-7 overexpression on predicting poor OS was found in the studies with NOS<7 (HR=2.43, 95% CI: 1.85-3.19, P<0.001) as well as those with NOS>7 (HR= 1.65, 95% CI: 1.11-2.45, P=0.013) (**Figure 3D**). And in studies published in 2018 and earlier, the pooled HR suggests that abnormally high expression of CDR1as/ciRS-7 is associated with poorer overall survival (HR= 2.20, 95% CI: 1.59-3.04, P<0.001). However, in studies published after 2018, our analysis did not replicate this association (HR= 1.30, 95% CI: 0.57-2.96, P=0.527) (**Figure 3E**).

**Figure 4:**
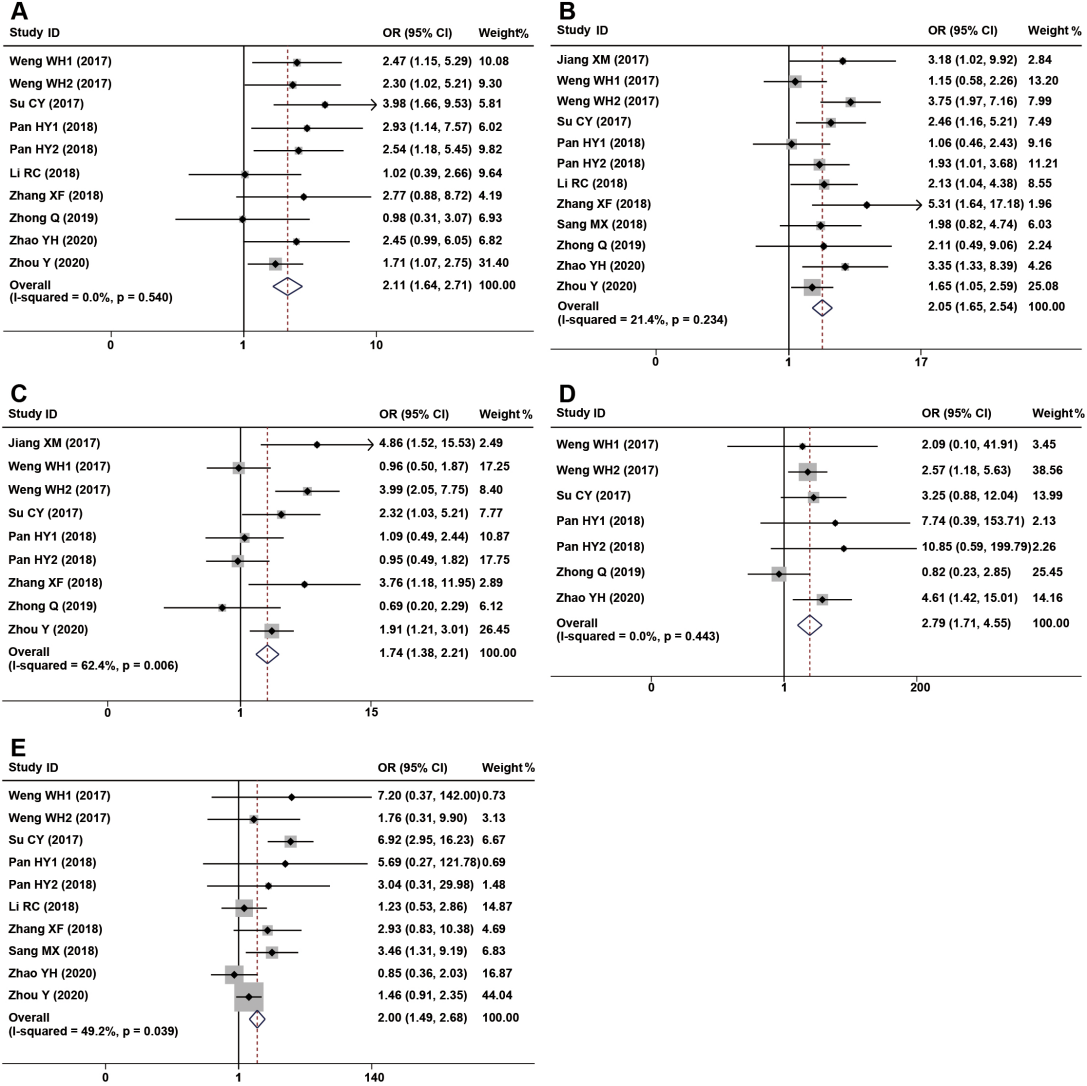
Forest plots of the included studies evaluating the correlation between CDR1as/ciRS-7 expression and clinicopathological characteristics. **(A)** tumor size; **(B)** TNM stages; **(C)** LNM; **(D)** distant metastases; **(E)** tumor differentiation.

The association between CDR1as/ciRS-7 expression and clinicopathological characteristics are examined in 12 studies with 2065 cancer patients (**Table 2**). 10 studies with 1372 cancer patients were included to analyze the correlation between CDR1as/ciRS-7 and tumor size, and the pooled data showed an obvious association between CDR1as/ciRS-7 and tumor size (OR=2.11, 95%CI: 1.64-2.71, P<0.001) (**Figure 4A**). The analysis results of 12 studies with 1518 cancer patients showed that there was a significant correlation between CDR1as/ciRS-7 and TNM stage (OR=2.05, 95%CI: 1.65-2.54, P<0.001) (**Figure 4B**). As indicated in **Figure 4C**, 1205 cancer patients from 9 studies were included to assess the association between CDR1as/ciRS-7 and LNM, and the results demonstrated that the patients with high CDR1as/ciRS-7 expression were more susceptibility to develop LNM (OR=1.74, 95%CI: 1.38-2.21, P<0.001). In addition, 7 studies with 780 cancer patients were included to analyze the association between CDR1as/ciRS-7 and distant metastasis. The results showed an obviously association between CDR1as/ciRS-7 expression and distant metastasis (OR=2.79, 95%CI: 1.71-4.55, P<0.001) (**Figure 4D**). We also analyzed the relationship between CDR1as/ciRS-7 and tumor differentiation using the data of 1380 cancer patients from 10 studies. The results showed CDR1as/ciRS-7 expression is also correlated with tumor differentiation (OR=2.00, 95%CI: 1.49-2.68, P<0.001) (**Figure 4E**). However, there was no significant correlation between CDR1as/ciRS-7 and other clinicopathological features, such as age (Z=0.01, P=0.990) and gender (Z=1.50, P=0.135).

### 3.3 Publication bias and sensitivity analysis

**Figure 5:**
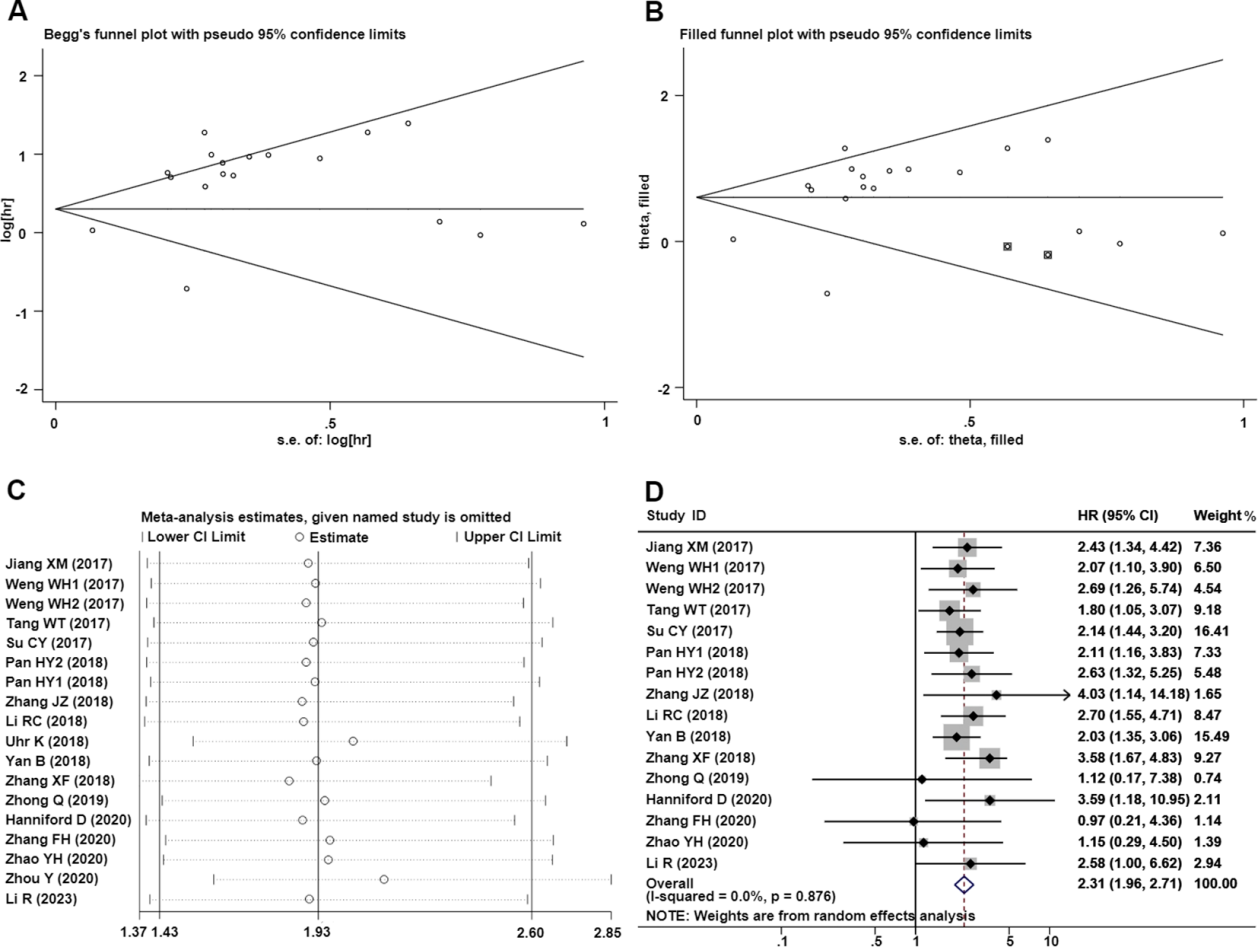
Publication bias and sensitivity analysis for OS in this meta-analysis. **(A)** Begg’s funnel plots of the included studies for OS; **(B)** Begg’s funnel plots of the included studies for OS after trim and fill analysis; **(C)** Sensitivity analysis of the included studies for OS; **(D)** Forest plots of the studies which remove Uhr et al. (26) and Zhou et al. (33).

To evaluate the publication bias, the Begg’s funnel plots and Egger’s linear regression tests were applied in this meta-analysis. According to the analysis of publication bias in our study, visual inspection of the Begg’s funnel plot revealed obvious asymmetry (**Figure 5A**), and Egger’s test suggested this study may get significant publication bias (t=3.02, P=0.008). Thus, to assess the impact of potential publication bias, the trim and fill analysis was performed with the random-effects model. Two which conservatively imputes hypo-thetical negative unpublished studies to mirror the positive studies that cause funnel plot asymmetry. The imputed studies produce a symmetrical funnel plot (**Figure 5B**). The pooled analysis incorporation the hypothetical studies continued to show a statistically significant association between CDR1as/ciRS-7 expression on OS in solid tumors (cor-rected HR=1.83, 95% CI: 1.38-2.43, P<0.001).

Significant heterogeneity was observed in sensitivity values (I²=80.2%, P<0.001), prompting further investigation into potential sources of interstudy heterogeneity. Meta-regression analysis revealed that none of the examined covariates, including publication year, sample size, NOS score, or region, significantly contributed to the observed heterogeneity (**Table 3**). And we also used sensitivity analysis to find the source of heterogeneity. It demonstrated that the pooled HR for the independent prognostic value of CDR1as/ciRS-7 in cancers was not significantly affected by the exclusion of any of the studies (**Figure 5C**). And according to the results of sensitivity analyzes the studies by Uhr K et al. (26) and Zhou Y et al. (33) were the top one with het-erogeneity in the OS group, but their removal changed the results into more significant ones with no heterogeneity (HR=2.31, 95% CI: 1.96-2.71,P<0.001; I2 = 0%, P=0.876) (**Figure 5D**).

**Table 3:** Meta-regression analysis of covariates contributing to interstudy heterogeneity.

Covariate	Coefficient	95% CI	Standard Error	p-value	I² Reduction
publication year	-0.100	-0.304 - 0.104	0.096	0.315	0.89%
sample size	-0.278	-0.926 - 0.370	0.306	0.377	3.7%
NOS score	0.399	-0.189 - 0.987	0.277	0.170	4.87%
region	-0.305	-1.185 - 0.574	0.415	0.473	12%

In addition, we also evaluated the association between CDR1as/ciRS-7 and tumor size, TNM stages, LNM, distant metastases and tumor differentiation. Visual inspection of the Begg’s funnel plots revealed symmetry in the studies investigating CDR1as/ciRS-7 on tumor size (t=0.39, P=0.707), TNM stages (t=1.28, P=0.228), LNM (t=0.25, P=0.81), distant metastases (t=0.72, P=0.502) and tumor differentiation (t=0.31, P=0.764), which suggested that there was no evidence of publication bias among the studies investigating CDR1as/ciRS-7 on tumor size, TNM stages, LNM, distant metastases and tumor differentiation.

## 4 Discusses

CircRNAs are endogenous non-coding RNAs with functions similar to lncRNAs. They have been indicated to play functions in the occurrence and development of cancer. Recent studies have shown that abnormal circRNAs expression in various types of solid tumors. That may make CircRNAs have diagnostic value. And targeting CircRNAs may sensitize the drug treatment, and it is a promising therapeutic target for cancer patients.

CDR1as/ciRS-7, as a newly discovered oncogene, is an important CircRNA in hu-man malignancies. Recent studies have found that CDR1as/ciRS-7 has multiple biological functions and is involved in cell proliferation, chemotherapy-resistance, invasion and metastasis, leading to the initialization and development of tumors. Comprehensive analysis indicates that in many solid tumors, abnormal expression of CDR1as/ciRS-7 is associated with clinical characteristics such as tumor size, tumor differentiation, TNM stage, lymph node metastasis, and distant metastasis (38–42). These results indicate that CDR1as/ciRS-7 plays a crucial role in the development and progression of cancer. Further exploration of its underlying mechanisms and potential clinical applications could provide novel insights for the diagnosis and treatment of various cancers. By understanding how CDR1as/ciRS-7 influences tumor biology, researchers may uncover new therapeutic strategies and biomarkers that enhance patient management and outcomes in oncology. However, due to heterogeneity, the perplexity and inconsistence conclusion exists in different studies. Thus, we conducted this meta-analysis to explore the clinicopathological and prognostic value of CDR1as/ciRS-7 as a potential biomarker in human solid tumors.

This comprehensive and systematic meta-analysis provides a holistic assessment of the association between the expression of CDR1as/ciRS-7 and cancer. By synthesizing data from 17 studies encompassing 2424 patients, the analysis revealed several key findings: (1) The elevating of CDR1as/ciRS-7 expression predicted shorter OS for 12 different solid tumors and was an independent predictor of patient prognosis. (2) CDR1as/ciRS-7 expression is significantly associated with several clinicopathological characteristics, including larger tumor size, advanced TNM stage, lymph node metastasis, distant metastasis, and tumor differentiation, but shows no significant correlation with age or gender. (3) The expression of CDR1as/ciRS-7 was inversely associated with poor prognosis in gynecologic cancer. Taken together, our results provide compelling evidence supporting CDR1as/ciRS-7 as a negative, adverse prognostic biomarker for the human solid tumors we analyzed. Of note, in gynecologic cancer, high expression of CDR1as/ciRS-7 is associated with favorable prognosis, which contrasts with our findings in other cancers. The results of this meta-analysis can inform the design of future clinical trials. And the abnormal expression of CDR1as/ciRS-7 may aid in diagnosis and treatment decision-making, suggesting its potential as a biomarker. Future studies could explore CDR1as/ciRS-7 as a therapeutic target with applications in personalized treatment.

Our analysis revealed significant heterogeneity in sensitivity values (I²=80.2%, P<0.001). Thus, we prompted an examination of potential sources through meta-regression and sensitivity analyses. Meta-regression indicated that covariates such as publication year, sample size, NOS score, and geographic region did not significantly account for this heterogeneity. Sensitivity analysis confirmed that the prognostic value of CDR1as/ciRS-7 in cancer was robust, with no individual study substantially impacting the pooled HR. Notably, after removing studies by Uhr et al. (26) and Zhou et al. (33) from the OS analysis, the heterogeneity markedly decreased (HR=2.31, 95% CI: 1.96-2.71, P<0.001; I²=0%, P=0.876), however the results remained similar to those before exclusion (HR=1.93, 95% CI: 1.43-2.60, P<0.001; I²=80.2%, P<0.001). It supports the stability and reliability of our findings. Additionally, funnel plot assessment showed no publication bias in our study, further supporting the reliability of these findings.

**Figure 6:**
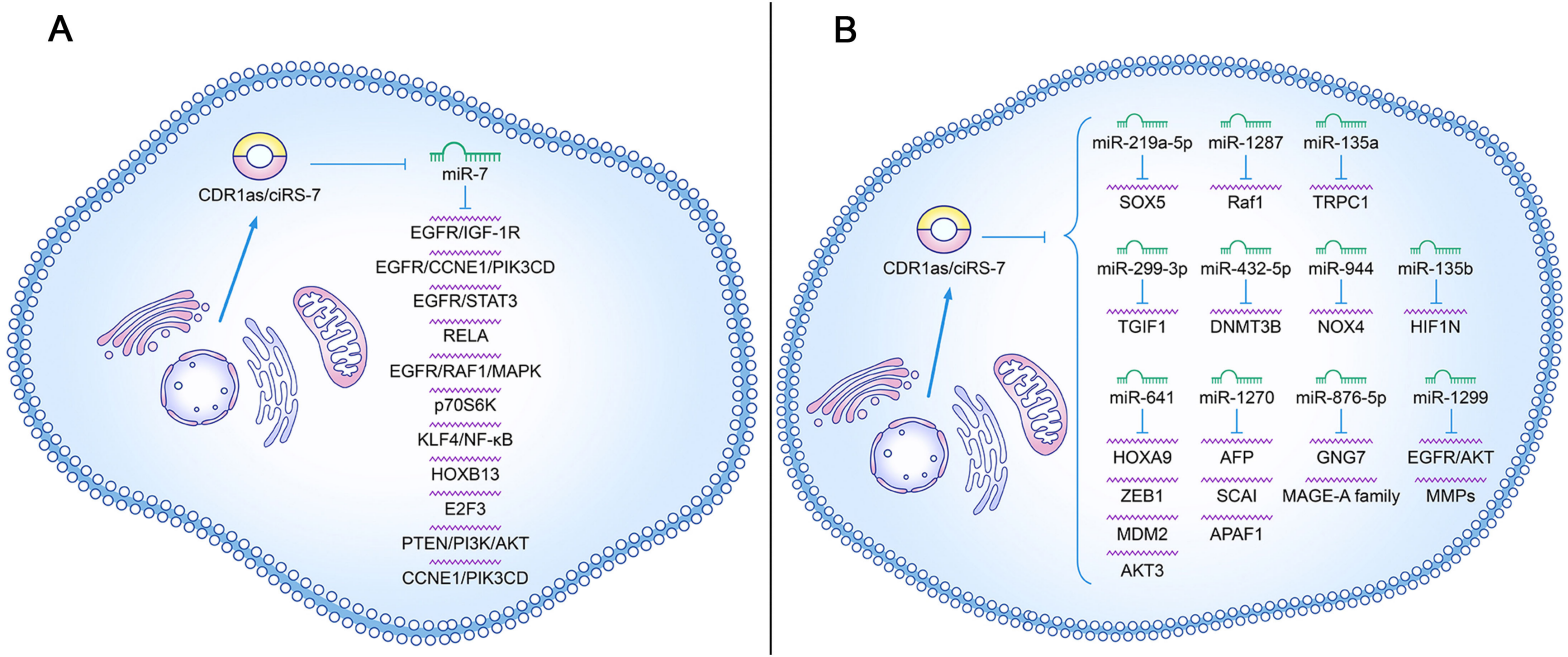
Roles of CDR1as/ciRS-7 in cancer initiation and progression. **(A)** The regulatory role of CDR1as/ciRS-7 in inhibiting miR-7 target gene signaling in tumor development; **(B)** The regulatory role of CDR1as/ciRS-7 in influencing signaling pathways independent of miR-7 targets in tumor development.

The underlying molecular mechanisms involved in CDR1as/ciRS-7 are complex and diverse in different cancers. CDR1as/ciRS-7 levels increased in multiple cancers, sponged miR-7, and then promoted the expression of downstream target genes, thereby playing a carcinogenic role (**Figure 6A**). NSCLC cell growth can be promoted by the overexpression of CDR1as/ciRS-7 which sponges miR-7 to upregulate target genes, such as EGFR, CCNE1, and PIK3CD (22). It was reported that CDR1as/ciRS-7 increased the proliferation, invasion, migration, and apoptosis of NSCLC cells through the miR-7/RELA axis (20). The levels of p70S6K mRNA and protein expression can be in-creased by the downregulation of miR-7, which may be correlated with microvascular invasion (MVI), younger age, and higher AFP level in HCC (43). One study showed that the level of CDR1as/ciRS-7 expression is increased in ESCC, and it is correlated with poor survival. Moreover, CDR1as/ciRS-7 sponges miR-7 to reactivate HOXB13-NF-κB/p56 signalling (44). CDR1as/ciRS-7 accelerates the invasion and migration of cells through miR-7-KLF4-NF-κB pathways in ESCC (45). CDR1as/ciRS-7 up-regulates the expression of E2F3 by binding miR-7-5p, which may promote the occurrence and development of NPC (30). The overexpression of CDR1as/ciRS-7 promotes an aggressive behaviour of GC cells by suppressing miR-7-involved PTEN/PI3K/AKT signalling (28). CDR1as/ciRS-7 upregulates CCNE1 and PIK3CD expression by sponging miR-7, and it may promote LSCC progression (23). CDR1as/ciRS-7 plays an oncogene role in PDAC, partly by targeting miR-7 and regulating the EGFR/STAT3 signalling pathway (46). Positive correlations between CDR1as/ciRS-7 expression and EGFR and IGF-1R expression were observed in CRC samples. Thus, given the importance of CDR1as/ciRS-7 in blocking miR-7 and positively regulating EGFR and IGF-1R, dysregulated CDR1as expression may play an important role in CRC progression (18). CDR1as/ciRS-7 overexpression permitted the inhibition of miR-7 and subsequent activation of EGFR and RAF1 oncogenes, which causes more aggressive oncogenic phenotype in CC cells (19).

In addition, CDR1as may also regulate other microRNAs to influence the progression of different cancers (**Figure 6B**). CDR1as/ciRS-7 also promotes the proliferation and migration of HCC cells by sponging miR-1270 to upregulate AFP expression (47). CDR1as/ciRS-7 enhances MMP expression to increase cellular invasion and migration in BC cells by acting as a ceRNA of miR-1299 (48). CDR1as/ciRS-7 acts as a sponge of miR-876-5p to increase MAGE-A family expression in ESCC cells, which promotes the progression of ESCC (25). Fur-ther research has reported that CDR1as/ciRS-7 sponged miR-1299 to inhibit ESCC cell autophagy by targeting the EGFR-AKT-mTOR pathway (49). Another study showed that CDR1as/ciRS-7 promotes the oncogenic behaviour of CCC cells through binding with miR-641 to activate the AKT3/mTOR signalling pathway (50). Zhang et al. demonstrated that Cdr1as sensitizes ovarian cancer to cisplatin by regulating the miR-1270/SCAI signalling pathway (51). One study verified that Cdr1as exerts a cisplatin-chemo sensitization effect on bladder cancer cells through the Cdr1as/miR-1270/APAF1 axis (52). CDR1as depletion inhibits HCC cell proliferation and metastasis by miR-1287/Raf1 and MEK/ERK pathways (53). A study from Zhao et al. identified that circRNA CDR1as regulated stemness and DDP chemoresistance in NSCLC cells by targeting the miR-641/HOXA9 axis (54). It was reported that targeting ciRS-7/miR-641/ZEB1 or ciRS-7/miR-641/MDM2 axis may be a novel diagnostic, prognostic, and therapeutic strategy for OC (31). Another study showed that knockdown of circCDR1as inhibited the progression of NSCLC by decreasing cell viability, migration, invasion and increasing apoptosis by upregulating miR-219a-5p and downregulating SOX5 (55). Jiang et al. found that CDR1as suppresses GC metastasis through the CDR1as/miR-876-5p/GNG7 axis (56). TRPC1 exacerbated EMT in gastric cancer via ciRS-7/miR-135a-5p/TRPC1 axis (57). Chen et al. showed that CDR1as acted as a sponge of miR-135b-5p, promotes the expression of HIF1AN and therefore plays a role in the inhibition of ovarian cancer (58). ciRS-7 promoted the progression of LSCC through increasing TGM3 methylation via miR-432-5p/DNMT3B axis (59). The article of Mao et al. demonstrated that ciRS-7 enhanced the proliferation, migration, and invasion of HCC through miR-944/NOX4 pathway (60).

Recently, there were many evidence showing that CDR1as/ciRS-7 functioned in a miRNA-independent manner. Hernando et al. reported that CDR1as/ciRS-7 cound interact with IGF2BP3 and sequester it from target mRNAs, that suppressed invasion and metastasis of IGF2BP3-mediated Melanoma (29). Recent studies suggest that ciRS-7 may sense DNA damage signals and preserve p53 tumor-suppressor function in glioma, highlighting a novel role in the DNA damage response (61). Wang et al. propose that ciRS-7 may enhance miRISC condensation, potentially promoting DNA double-strand break repair via AGO2-mediated homologous recombination (62).

While numerous studies have explored the mechanisms of CDR1as/ciRS-7 across various cancers, to our knowledge, its potential as a therapeutic target and biomarker has yet to be translated into clinical diagnostic or therapeutic applications. Additionally, we found no in-depth studies on the unusually low expression of ciRS-7 in gynecologic cancers, which contrasts with findings in other tumor types in this meta-analysis.

Some limitations exist in this meta-analysis. At first, the data across these clinical studies is discrete. Second the definitions of the cutoff values of CDR1as/ciRS-7 expression level in different studies are not the same. Third, we only analyzed the reported HR or survival curves of these studies, which may lead to the potential for selection bias. Moreover, this study is limited due to the following reasons: some of the HRs and CIs were calculated by the Kaplan-Meier curves, sample size is different, and the survival rate were chosen at a specified time. Hence, a calculation bias might exist. In addition, the data collection may be incomplete as we the language of the involved studies was limited to English. Furthermore, in this meta-analysis, most of the included studies reported positive results, and the summarized data rather than individual patient data were used. Therefore, it is possible that our results might overestimate the effects of abnormal CDR1as/ciRS-7 expression on survival and clinical characteristics in different types of cancers. These factors may contribute to heterogeneity and publication bias in this meta-analysis, potentially affecting the credibility of the results. To address this, we applied subgroup analysis, sensitivity analysis, and meta-regression to investigate sources of heterogeneity. Additionally, we implemented strict inclusion criteria to ensure similarity across studies, which can help reduce heterogeneity. To assess publication bias, we employed funnel plots and Egger’s test. Together, these methods effectively identify, assess, and control for limitations, thereby enhancing the reliability of the meta-analysis findings.

## 5 Conclusions

In summary, this meta-analysis suggested that the expression of CDR1as/ciRS-7 was correlated with the prognosis of cancer patients. Our results showed that the abnormal expression of CDR1as/ciRS-7 was significantly correlated with the differentiation of tumors, TNM stage, lymph node metastasis, distant metastasis, and other clinicopathological factors. Therefore, CDR1as/ciRS-7 can be used as a new biomarker for the prognosis of patients with cancers.

## Data Availability

The original contributions presented in the study are included in the article/supplementary material. Further inquiries can be directed to the corresponding authors.

## References

[1] Siegel RL Miller KD Fuchs HE Jemal A . Cancer statistic. CA Cancer J Clin. (2021) 71:7–33. doi: 10.3322/caac.21654 33433946

[2] Chen W Zheng R Baade PD Zhang S Zeng H Bray F . Cancer statistics in China. CA Cancer J Clin. (2016) 66:115–32. doi: 10.3322/caac.21338 26808342

[3] Enuka Y Lauriola M Feldman ME Sas-Chen A Ulitsky I Yarden Y . Circular RNAs are long-lived and display only minimal early alterations in response to a growth factor. Nucleic Acids Res. (2016) 44:1370–83. doi: 10.1093/nar/gkv1367 PMC4756822 26657629

[4] Cao W Chen HD Yu YW Li N Chen WQ . Changing profiles of cancer burden worldwide and in China: a secondary analysis of the global cancer statistics 2020. Chin (Engl). (2021) 134(7):783–91. doi: 10.1097/CM9.0000000000001474 PMC8104205 33734139

[5] Gao J Xu W Wang J Wang K Li P . The role and molecular mechanism of non-coding RNAs in pathological cardiac remodeling. Int J Mol Sci. (2017) 18(3):608. doi: 10.3390/ijms18030608 28287427 PMC5372624

[6] Geng Y Jiang J Wu C . Function and clinical significance of circRNAs in solid tumors. J Hematol Oncol. (2018) 11:98. doi: 10.1186/s13045-018-0643-z 30064463 PMC6069963

[7] Greene J Baird AM Brady L Lim M Gray SG McDermott R . Circular RNAs: biogenesis, function and role in human diseases. Front Mol Biosci. (2017) 4:38. doi: 10.3389/fmolb.2017.00038 28634583 PMC5459888

[8] Kristensen LS Hansen TB Veno MT Kjems J . Circular RNAs in cancer: opportunities and challenges in the field. Oncogene. (2018) 37:555–65. doi: 10.1038/onc.2017.361 PMC5799710 28991235

[9] Qu S Yang X Li X Wang J Gao Y Shang R . Circular RNA: A new star of noncoding RNAs. Cancer Lett. (2015) 365:141–8. doi: 10.1016/j.canlet.2015.06.003 26052092

[10] Zhang X Lu J Zhang Q Luo Q Liu B . CircRNA RSF1 regulated ox-LDL induced vascular endothelial cells proliferation, apoptosis and inflammation through modulating miR-135b-5p/HDAC1 axis in atherosclerosis. Biol Res. (2021) 54:11. doi: 10.1186/s40659-021-00335-5 33757583 PMC7986494

[11] Yang Y Liu L Tian Y Gu M Wang Y Ashrafizadeh M . Autophagy-driven regulation of cisplatin response in human cancers: Exploring molecular and cell death dynamics. Cancer Lett. (2024) 587:216659. doi: 10.1016/j.canlet.2024.216659 38367897

[12] Barrett SP Parker KR Horn C Mata M Salzman J . ciRS-7 exonic sequence is embedded in a long non-coding RNA locus. PloS Genet. (2017) 13:e1007114. doi: 10.1371/journal.pgen.1007114 29236709 PMC5745005

[13] Hansen TB Jensen TI Clausen BH Bramsen JB Finsen B Damgaard CK . Natural RNA circles function as efficient microRNA sponges. Nature. (2013) 495:384–8. doi: 10.1038/nature11993 23446346

[14] Memczak S Jens M Elefsinioti A Torti F Krueger J Rybak A . Circular RNAs are a large class of animal RNAs with regulatory potency. Nature. (2013) 495:333–8. doi: 10.1038/nature11928 23446348

[15] Hansen TB Kjems J Damgaard CK . Circular RNA and miR-7 in cancer. Cancer Res. (2013) 73:5609–12. doi: 10.1158/0008-5472.CAN-13-1568 24014594

[16] Peng L Yuan XQ Li GC . The emerging landscape of circular RNA ciRS-7 in cancer (Review). Oncol Rep. (2015) 33:2669–74. doi: 10.3892/or.2015.3904 25873049

[17] Jiang XM Li ZL Li JL Xu Y Leng KM Cui YF . A novel prognostic biomarker for cholangiocarcinoma: circRNA Cdr1as. Eur Rev Med Pharmacol Sci. (2018) 22:365–71. doi: 10.26355/eurrev20180114182 29424892

[18] Tang W Ji M He G Yang L Niu Z Jian M . Silencing CDR1as inhibits colorectal cancer progression through regulating microRNA-7. Onco Targets Ther. (2017) 10:2045–56. doi: 10.2147/OTT.S131597 PMC5391170 28435295

[19] Weng W Wei Q Toden S Yoshida K Nagasaka T Fujiwara T . Circular RNA ciRS-7-A promising prognostic biomarker and a potential therapeutic target in colorectal cancer. Clin Cancer Res. (2017) 23:3918–28. doi: 10.1158/1078-0432.CCR-16-2541 PMC5511556 28174233

[20] Su C Han Y Zhang H Li Y Yi L Wang X . CiRS-7 targeting miR-7 modulates the progression of non-small cell lung cancer in a manner dependent on NF-kappaB signalling. J Cell Mol Med. (2018) 22:3097–107. doi: 10.1111/jcmm.2018.22.issue-6 PMC5980210 29532994

[21] Yan B Zhang W Mao XW Jiang LY . Circular RNA ciRS-7 correlates with advance disease and poor prognosis, and its down-regulation inhibits cells proliferation while induces cells apoptosis in non-small cell lung cancer. Eur Rev Med Pharmacol Sci. (2018) 22:8712–21. doi: 10.26355/eurrev20181216636 30575911

[22] Zhang X Yang D Wei Y . Overexpressed CDR1as functions as an oncogene to promote the tumor progression via miR-7 in non-small-cell lung cancer. Onco Targets Ther. (2018) 11:3979–87. doi: 10.2147/OTT.S158316 PMC6044366 30022841

[23] Zhang J Hu H Zhao Y Zhao Y . CDR1as is overexpressed in laryngeal squamous cell carcinoma to promote the tumour's progression via miR-7 signals. Cell Prolif. (2018) 51:e12521. doi: 10.1111/cpr.2018.51.issue-6 30182381 PMC6528957

[24] Li RC Ke S Meng FK Lu J Zou XJ He ZG . CiRS-7 promotes growth and metastasis of esophageal squamous cell carcinoma via regulation of miR-7/HOXB13. Cell Death Dis. (2018) 9:838. doi: 10.1038/s41419-018-0852-y 30082829 PMC6079012

[25] Sang M Meng L Sang Y Liu S Ding P Ju Y . Circular RNA ciRS-7 accelerates ESCC progression through acting as a miR-876-5p sponge to enhance MAGE-A family expression. Cancer Lett. (2018) 426:37–46. doi: 10.1016/j.canlet.2018.03.049 29635069

[26] Uhr K Sieuwerts AM de Weerd V Smid M Hammerl D Foekens JA . Association of microRNA-7 and its binding partner CDR1-AS with the prognosis and prediction of 1(st)-line tamoxifen therapy in breast cancer. Sci Rep. (2018) 8(1):9657. doi: 10.1038/s41598-018-27987-w 29941867 PMC6018428

[27] Li R Tian X Jiang J Qian H Shen H Xu W . CircRNA CDR1as: a novel diagnostic and prognostic biomarker for gastric cancer. Biomarkers. (2023) 28:448–57. doi: 10.1080/1354750X.2023.2206984 37128800

[28] Pan H Li T Jiang Y Pan C Ding Y Huang Z . Overexpression of Circular RNA ciRS-7 Abrogates the Tumor Suppressive Effect of miR-7 on Gastric Cancer via PTEN/PI3K/AKT Signaling Pathway. J Cell Biochem. (2018) 119:440–46. doi: 10.1002/jcb.v119.1 28608528

[29] Hanniford D Ulloa-Morales A Karz A Berzoti-Coelho MG Moubarak RS Sanchez-Sendra B . Epigenetic silencing of CDR1as drives IGF2BP3-mediated melanoma invasion and metastasis. Cancer Cell. (2020) 37 55-70:e15. doi: 10.1016/j.ccell.2019.12.007 31935372 PMC7184928

[30] Zhong Q Huang J Wei J Wu R . Circular RNA CDR1as sponges miR-7-5p to enhance E2F3 stability and promote the growth of nasopharyngeal carcinoma. Cancer Cell Int. (2019) 19:252. doi: 10.1186/s12935-019-0959-y 31582908 PMC6771089

[31] Zhang F Xu Y Ye W Jiang J Wu C . Circular RNA S-7 promotes ovarian cancer EMT via sponging miR-641 to up-regulate ZEB1 and MDM2. Biosci Rep. (2020) 40(7):BSR20200825. doi: 10.1042/BSR20200825 32667627 PMC7383824

[32] Zhao YH Wang Z Zhang N Cui T Zhang YH . Effect of ciRS-7 expression on clear cell renal cell carcinoma progression. Chin (Engl). (2020) 133(17):2084–9. doi: 10.1097/CM9.0000000000000867 PMC7478654 32496306

[33] Zhou Y Shen L Wang YZ Zhou CC . The potential of ciRS-7 for predicting onset and prognosis of cervical cancer. Neoplasma. (2020) 67:312–22. doi: 10.4149/neo_2019_190415N334 31884800

[34] Azari H Mousavi P Karimi E Sadri F Zarei M Rafat M . The expanding role of CDR1-AS in the regulation and development of cancer and human diseases. J Cell Physiol. (2021) 236:771–90. doi: 10.1002/jcp.v236.2 32697389

[35] Chen J Yang J Fei X Wang X Wang K . CircRNA ciRS-7: a novel oncogene in multiple cancers. Int J Biol Sci. (2021) 17:379–89. doi: 10.7150/ijbs.54292 PMC7757028 33390857

[36] Jiang C Zeng X Shan R Wen W Li J Tan J . The emerging picture of the roles of circRNA-CDR1as in cancer. Front Cell Dev Biol. (2020) 8:590478. doi: 10.3389/fcell.2020.590478 33335899 PMC7736612

[37] Tang X Ren H Guo M Qian J Yang Y Gu C . Review on circular RNAs and new insights into their roles in cancer. Comput Struct Biotechnol J. (2021) 19:910–28. doi: 10.1016/j.csbj.2021.01.018 PMC7851342 33598105

[38] Chen G Wang Q Li Z Yang Q Liu Y Du Z . Circular RNA CDR1as promotes adipogenic and suppresses osteogenic differentiation of BMSCs in steroid-induced osteonecrosis of the femoral head. Bone. (2020) 133:115258. doi: 10.1016/j.bone.2020.115258 32018039

[39] Wang F Chen X Han Y Xi S Wu G . circRNA CDR1as regulated the proliferation of human periodontal ligament stem cells under a lipopolysaccharide-induced inflammatory condition. Mediators Inflammation. (2019) 2019:1625381. doi: 10.1155/2019/1625381 PMC6754938 31582895

[40] Yang L Bin Z Hui S Rong L You B Wu P . The role of CDR1as in proliferation and differentiation of human umbilical cord-derived mesenchymal stem cells. Stem Cells Int. (2019) 2019:2316834. doi: 10.1155/2019/2316834 31281369 PMC6594288

[41] Yang X Li S Wu Y Ge F Chen Y Xiong Q . The circular RNA CDR1as regulate cell proliferation via TMED2 and TMED10. BMC Cancer. (2020) 20:312. doi: 10.1186/s12885-020-06794-5 32293333 PMC7160961

[42] Zhao F Chen T Jiang N . CDR1as/miR-7/CKAP4 axis contributes to the pathogenesis of abdominal aortic aneurysm by regulating the proliferation and apoptosis of primary vascular smooth muscle cells. Exp Ther Med. (2020) 19:3760–66. doi: 10.3892/etm.2020.8622 PMC7185088 32346440

[43] Xu L Zhang M Zheng X Yi P Lan C Xu M . The circular RNA ciRS-7 (Cdr1as) acts as a risk factor of hepatic microvascular invasion in hepatocellular carcinoma. J Cancer Res Clin Oncol. (2017) 143:17–27. doi: 10.1007/s00432-016-2256-7 27614453 PMC11819007

[44] Yang W Gu J Wang X Wang Y Feng M Zhou D . Inhibition of circular RNA CDR1as increases chemosensitivity of 5-FU-resistant BC cells through up-regulating miR-7. J Cell Mol Med. (2019) 23:3166–77. doi: 10.1111/jcmm.2019.23.issue-5 PMC6484300 30884120

[45] Huang H Wei L Qin T Yang N Li Z Xu Z . Circular RNA ciRS-7 triggers the migration and invasion of esophageal squamous cell carcinoma via miR-7/KLF4 and NF-kappaB signals. Cancer Biol Ther. (2019) 20:73–80. doi: 10.1080/15384047.2018.1507254 30207835 PMC6343722

[46] Liu L Liu FB Huang M Xie K Xie QS Liu CH . Circular RNA ciRS-7 promotes the proliferation and metastasis of pancreatic cancer by regulating miR-7-mediated EGFR/STAT3 signaling pathway. Hepatobiliary Pancreat Dis Int. (2019) 18:580–86. doi: 10.1016/j.hbpd.2019.03.003 30898507

[47] Su Y Lv X Yin W Zhou L Hu Y Zhou A . CircRNA Cdr1as functions as a competitive endogenous RNA to promote hepatocellular carcinoma progression. Aging (Albany NY). (2019) 11:8183–203. doi: 10.18632/aging.102312 PMC6814590 31581132

[48] Sang M Meng L Liu S Ding P Chang S Ju Y . Circular RNA ciRS-7 Maintains Metastatic Phenotypes as a ceRNA of miR-1299 to Target MMPs. Mol Cancer Res. (2018) 16:1665–75. doi: 10.1158/1541-7786.MCR-18-0284 30072582

[49] Meng L Liu S Ding P Chang S Sang M . Circular RNA ciRS-7 inhibits autophagy of ESCC cells by functioning as miR-1299 sponge to target EGFR signaling. J Cell Biochem. (2020) 121:1039–49. doi: 10.1002/jcb.v121.2 31490018

[50] Li D Tang Z Gao Z Shen P Liu Z Dang X . Circular RNA CDR1as exerts oncogenic properties partially through regulating microRNA 641 in cholangiocarcinoma. Mol Cell Biol. (2020) 40(15):e00042–20. doi: 10.1128/MCB.00042-20 PMC7364046 32423991

[51] Zhao Z Ji M Wang Q He N Li Y . Circular RNA Cdr1as Upregulates SCAI to Suppress Cisplatin Resistance in Ovarian Cancer via miR-1270 Suppression. Mol Ther Nucleic Acids. (2019) 18:24–33. doi: 10.1016/j.omtn.2019.07.012 31479922 PMC6726918

[52] Yuan W Zhou R Wang J Han J Yang X Yu H . Circular RNA Cdr1as sensitizes bladder cancer to cisplatin by upregulating APAF1 expression through miR-1270 inhibition. Mol Oncol. (2019) 13:1559–76. doi: 10.1002/mol2.2019.13.issue-7 PMC6599840 31131537

[53] Zhang B Li F Zhu Z Ding A Luo J . CircRNA CDR1as/miR-1287/raf1 axis modulates hepatocellular carcinoma progression through MEK/ERK pathway. Cancer Manag Res. (2020) 12:8951–64. doi: 10.2147/CMAR.S252679 PMC7522432 33061591

[54] Zhao Y Zheng R Chen J Ning D . CircRNA CDR1as/miR-641/HOXA9 pathway regulated stemness contributes to cisplatin resistance in non-small cell lung cancer (NSCLC). Cancer Cell Int. (2020) 20:289. doi: 10.1186/s12935-020-01390-w 32655321 PMC7339514

[55] Li Y Zhang J Pan S Zhou J Diao X Liu S . CircRNA CDR1as knockdown inhibits progression of non-small-cell lung cancer by regulating miR-219a-5p/SOX5 axis. Thorac Cancer. (2020) 11:537–48. doi: 10.1111/1759-7714.13274 PMC7049501 31917898

[56] Jiang J Li R Wang J Hou J Qian H Xu W . Circular RNA CDR1as Inhibits the Metastasis of Gastric Cancer through Targeting miR-876-5p/GNG7 Axis. Gastroenterol Res Pract. (2021) 2021:5583029. doi: 10.1155/2021/5583029 34221006 PMC8225434

[57] Zhang Z Ren L Zhao Q Lu G Ren M Lu X . TRPC1 exacerbate metastasis in gastric cancer via ciRS-7/miR-135a-5p/TRPC1 axis. Biochem Biophys Res Commun. (2020) 529:85–90. doi: 10.1016/j.bbrc.2020.05.181 32560824

[58] Chen H Mao M Jiang J Zhu D Li P . Circular RNA CDR1as acts as a sponge of miR-135b-5p to suppress ovarian cancer progression. Onco Targets Ther. (2019) 12:3869–79. doi: 10.2147/OTT.S207938 PMC6529026 31190886

[59] Zhao K Ye F Gao P Zhu X Hao S Lou W . Circular RNA ciRS-7 promotes laryngeal squamous cell carcinoma development by inducing TGM3 hypermethylation via miR-432-5p/DNMT3B axis. Pathol Res Pract. (2022) 240:154193. doi: 10.1016/j.prp.2022.154193 36356335

[60] Mao C Wen H Zhang Y Yu G Ge Q . ciRS-7 Enhances the Progression of Hepatocellular Carcinoma through miR-944/NOX4 Pathway. Crit Rev Eukaryot Gene Expr. (2022) 32:11–24. doi: 10.1615/CritRevEukaryotGeneExpr.2022039225 36004692

[61] Lou J Hao Y Lin K Lyu Y Chen M Wang H . Circular RNA CDR1as disrupts the p53/MDM2 complex to inhibit Gliomagenesis. Mol Cancer. (2020) 19:138. doi: 10.1186/s12943-020-01253-y 32894144 PMC7487905

[62] Wang YL Feng LL Shi J Chen WY Bie SY Bai SM . CiRS-7 enhances the liquid-liquid phase separation of miRISC and promotes DNA damage repair. Nucleus. (2023) 14:2293599. doi: 10.1080/19491034.2023.2293599 38105528 PMC10730229

